# Self-reported olfactory and gustatory dysfunctions in COVID-19 patients: a 1-year follow-up study in Foggia district, Italy

**DOI:** 10.1186/s12879-022-07052-8

**Published:** 2022-01-22

**Authors:** Francesca Fortunato, Domenico Martinelli, Giuseppina Iannelli, Marica Milazzo, Umberto Farina, Gabriella Di Matteo, Rosella De Nittis, Leonardo Ascatigno, Michele Cassano, Pier Luigi Lopalco, Rosa Prato

**Affiliations:** 1 Hygiene Unit, Policlinico Riuniti Foggia Hospital, Department of Medical and Surgical Sciences, University of Foggia, Ospedale Colonnello D’Avanzo, Viale degli Aviatori 2, 71122 Foggia, Italy; 2 Microbiology and Virology Section, Policlinico Riuniti Foggia Hospital, Foggia, Italy; 3 Otolaryngology - Head and Neck Surgery Unit, Policlinico Riuniti Foggia Hospital, Department of Clinical and Experimental Medicine, University of Foggia, Foggia, Italy; 4 Department of Biological and Environmental Sciences and Technology, University of Salento, Lecce, Italy

**Keywords:** Anosmia, Ageusia, Sensory dysfunctions, COVID-19, Prevalence, Recovery, Long COVID, Italy

## Abstract

**Background:**

From the initial stages of the pandemic in early 2020, COVID-19-related olfactory and gustatory dysfunctions have been widely reported and are emerging as one of the most frequent long-term sequelae of SARS-CoV-2 infection. However, data regarding the long-term recovery of the sense of smell and taste are lacking. This study aimed to characterize the evolution up to one year after the diagnosis of self-reported olfactory and gustatory dysfunctions in COVID-19 cases.

**Methods:**

Based on the data of the active surveillance platform of the Apulia region, Italy, we selected the residents of Foggia district who were confirmed positive for SARS-CoV-2 from March 1st to June 16th, 2020, and home-quarantined with paucisymptomatic-to-mild clinical presentation. Self-reported olfactory and gustatory dysfunctions were recorded at baseline through a survey of dichotomous questions. The evolution of these symptoms at approximately one year was prospectively assessed via telephone by the validated sino-nasal outcome test 22 (SNOT-22, Italian version).

**Results:**

Among the 1,175 COVID-19 cases notified in the Foggia district during the first epidemic wave, 488 had paucisymptomatic-to-mild clinical presentation. Of these, 41.2% (n = 201, 95% confidence interval [CI] 36.8–45.7%) reported at least one sensory dysfunction. A total of 178 to 201 (88.5%) patients agreed to participate in the follow-up survey. According to the SNOT-22 results, the persistence of a sensory dysfunction was observed in the 29.8% (n = 53, 95% CI 23.2–37.1%) of them. Particularly, loss of smell persisted in 25.8% (n = 46, 95% CI 19.6–32.9%), loss of taste in 21.3% (n = 38, 95% CI 15.6–28.1%), loss of both in 17.4% (n = 31, 95% CI 12.2–23.8%) of participants in the follow-up. The rates of full recovery increased over time: from 59% at 30 days to 71.9% at 90 days for the sense of smell; from 61.3% at 30 days to 74.7% at 90 days for the sense of taste.

**Conclusions:**

The persistence of COVID-19-related olfactory and gustatory dysfunctions up to 12 months after the disease onset in a noteworthy proportion (approximately 3 out of 10) of patients with paucisymptomatic-to-mild clinical presentation deserves further investigations due to its possible pathophysiological implications and impact on the quality of life.

**Supplementary Information:**

The online version contains supplementary material available at 10.1186/s12879-022-07052-8.

## Background

From the earliest stages of the coronavirus disease 19 (COVID-19) pandemic, in March 2020, the American Academy of Otolaryngology—Head and Neck Surgery proposed including anosmia and dysgeusia to the list of screening tools for possible COVID-19 infection [1]. In April 2020, the Centers for Disease Control and Prevention added ‘‘new loss of taste or smell’’ to the list of symptoms that may appear 2 to 14 days after exposure to the severe acute respiratory syndrome coronavirus 2 (SARS-CoV-2) [2]. As of December 3^rd^, 2020, the European Centre for Disease Prevention and Control included the “sudden onset of anosmia, ageusia or dysgeusia” among the clinical criteria of the new case definition for COVID-19 [3].

To date, several studies have documented a high prevalence of olfactory and gustatory dysfunctions in COVID-19. These sensory dysfunctions represent specific and sometimes early-onset symptoms, which differentiate SARS-CoV-2 infection from other acute respiratory diseases, mainly in paucisymptomatic patients [4–12].

The pathophysiological mechanism through which SARS-CoV-2 causes sensory dysfunctions of various severity has not been fully clarified yet. It is well known that SARS-CoV-2 infects alveolar epithelial cells by directly binding the Angiotensin-Converting Enzyme 2 (ACE2) cell receptors, thereby decreasing their expression and protective function. ACE2 receptors are expressed by cells of the gut, kidney, heart, and oral mucosa. In particular, they are highly expressed in the tongue compared with other areas of the oral cavity (vestibular and gingival), suggesting a peculiar vulnerability of the oral mucosa to COVID-19 infection [13]. Furthermore, anosmia could be ascribed to the involvement of the central nervous system and damage of the nasal epithelium. Indeed, a potential neuroinvasiveness of coronaviruses through the olfactory nerve pathway or the peripheral trigeminal endings has been described [6, 14]. Accordingly, SARS-CoV-2 RNA has been isolated in the cerebrospinal fluid [6, 15]. More recently, in patients who have recovered from COVID-19, neuroimaging has revealed localized inflammation in intracranial olfactory structures with alterations of primary olfactory neurons putatively leading to a persistent impairment [16]. Gupta et al. [17] suggested that loss of smell in SARS-CoV-2 infection is probably due to the susceptibility of a subgroup of cells (i.e., sustentacular cells, Bowman’s cells, and olfactory stem cells) to virus entry, rather than to the direct impairment of the olfactory sensory neurons. Sustentacular cells, Bowman’s cells and olfactory stem cells do not have a sensory function but play a crucial role in the maintenance of the olfactory organ and its homeostasis. Recent evidence from combined investigations of COVID-19-associated olfactory dysfunction in humans and experimentally-infected animals suggests that multiple cell types of the olfactory neuroepithelium are infected during the acute phase and that protracted viral infection and inflammation in the olfactory neuroepithelium may account for prolonged anosmia [18, 19].

In most cases, olfactory and gustatory dysfunctions resolve naturally and completely in the short term. However, several studies have demonstrated that moderate-to-severe olfactory or gustatory disturbances persist in a significant proportion of patients even months after the clinical onset of COVID-19 [20–29]. This observation is in agreement with the long-term sequelae referred to as “Long COVID”, which includes smell/taste disorders and chronic fatigue, among a variety of symptoms [30].

In this context, limited studies on the duration of olfactory and gustatory dysfunctions are available to date, and they predominantly document the mid-term evolution of these symptoms. Indeed, only a few studies have exceeded a 6-month follow-up and investigated the recovery of olfactory and gustatory dysfunctions beyond this period providing long-term information [21–23, 27, 28]. Nevertheless, considering the large number of people affected worldwide by COVID-19, the definition of duration and persistence of olfactory and gustatory disorders deserve to be addressed because of their potential impact on the quality of life [31].

This study aimed to characterize the long-term evolution of self-reported olfactory and gustatory dysfunctions in COVID-19 cases with previous paucisymptomatic-to-mild clinical presentation, estimating their persistence and recovery rate one year after the diagnosis.

## Methods

### Design, setting and participants

This was a prospective study conducted in the district of Foggia (Apulia region, Italy) between March 1st, 2020, when the first case of SARS-CoV-2 infection was confirmed by molecular testing, and June 16th, 2021.

During the first phase of the COVID-19 epidemic (March 1st—June 16th, 2020), all residents in the Foggia district testing positive for SARS-CoV-2 and home-quarantined with paucisymptomatic-to-mild clinical presentation were considered eligible for the initial survey. According to the definition of clinical status adopted by the “Integrated Surveillance of COVID-19 cases” of the National Institute of Health (Istituto Superiore di Sanità), paucisymptomatic referred to a COVID-19 case with generally mild symptoms but no clear signs of disease whereas mild referred to a COVID-19 case with clear signs and symptoms of disease which, however, were not severe enough to require hospitalization.

Residents in long-stay residential care homes were excluded.

### Data sources

Cases’ demographic and clinical characteristics were collected through the regional information system for health emergency management (Sistema informativo regionale per la gestione dell’emergenza sanitaria) named “GIAVA-COVID©”. GIAVA-COVID© was an active surveillance platform with twice-daily follow-up of cases until complete recovery with viral RNA clearance from the upper respiratory tract (two serial negative PCR tests at least 24 h apart) [32]. At the time of diagnosis, participants were contacted via telephone to answer four non-validated dichotomous questions. Subjects were asked to self-report subjective loss of smell and taste functions (Yes/No) and temporally relate its onset to that of other COVID-19 symptoms (fever, rhinorrhea, nasal congestion, cough, dyspnea; Yes/No). A quantitative assessment of the dysfunctions was not performed at this stage of the study [21, 25, 33].

Participants who had reported chemosensory dysfunctions at the time of the first questionnaire were contacted via telephone for a follow-up interview approximately 12 months after the study entry. In case of missed response, subjects were recontacted twice over fourteen days to minimize the possibility of a response bias. Loss of the sense of smell and/or taste was assessed retrospectively at the time of the follow-up interview in terms of duration (from the onset to the resolution dates, days) and severity according to sino-nasal outcome test 22 (SNOT-22), item “sense of smell or taste”. The SNOT-22 grades symptom severity as none (0), very mild (1), mild or slight (2), moderate (3), severe (4), or as bad as it can be (5) (SNOT-22, Italian version) [9, 34, 35]. If SNOT-22 > 0, participants were also asked whether the chemosensory dysfunction involved the sense of smell, taste, or both.

### Variables and definitions

The prevalence of olfactory and gustatory dysfunctions at baseline was calculated as the ratio of COVID-19 cases with self-reported impairment or loss of smell and/or taste to the total number of COVID-19 cases. The persistence of these symptoms at follow-up was calculated as the ratio of COVID-19 cases with persistent olfactory and gustatory dysfunctions on the date of the follow-up interview to the total number of participants in the follow-up phase. Complete recovery was defined as self-reported full resolution of smell and/or taste dysfunctions, whereas partial recovery was defined as an improvement in olfactory and/or gustatory functions [25]. The duration of chemosensory dysfunctions was calculated from the onset to the resolution dates of each symptom. If patients reported unresolved symptoms (no resolution date), the symptoms duration was registered at 365 days, as this was the longest calculated duration [29].

The number of incident cases in the Apulia region between March 1st and June 16th, 2020, determined the sample size.

### Statistical analysis

Descriptive statistics were performed. Categorical variables (sex, age group, comorbidities, clinical presentation) were expressed as counts and percentages in each category, continuous variables (age, incubation period, time to viral clearance, duration of chemosensory dysfunctions) as means (± standard deviation [SD]) and medians (interquartile ranges [IQR]). The prevalence, the persistence and the recovery rate of olfactory and gustatory dysfunctions were expressed as percentage with 95% confidence interval (CI) using Clopper-Pearson method [22].

The association between self-reported presence of sensory alterations at baseline and putatively related variables in the study group was assessed computing the chi-square test (*χ*^2^) and the odds ratio (ORs) with 95% CIs. Differences in continuous variables were tested with Student’s t test for normally distributed ones, or the Mann–Whitney *U* test when variables showed a non-normal distribution.

Multivariate logistic regression analysis was performed to evaluate whether demographics (sex: male vs. female; age group: above vs. below the median age), clinical characteristics (presence vs. absence of at least one underlying medical condition) and clinical presentation (paucisymptomatic vs. mild) were independently associated with baseline sensory dysfunctions and complete recovery.

Moreover, Kaplan–Meier estimates were reported to show the rates of complete recovery from the onset of the smell and/or taste loss [25].

The level of statistical significance was set at *p* ≤ 0.05.

Analysis was conducted with STATA/SE 15.0.

## Results

**Fig. 1:**
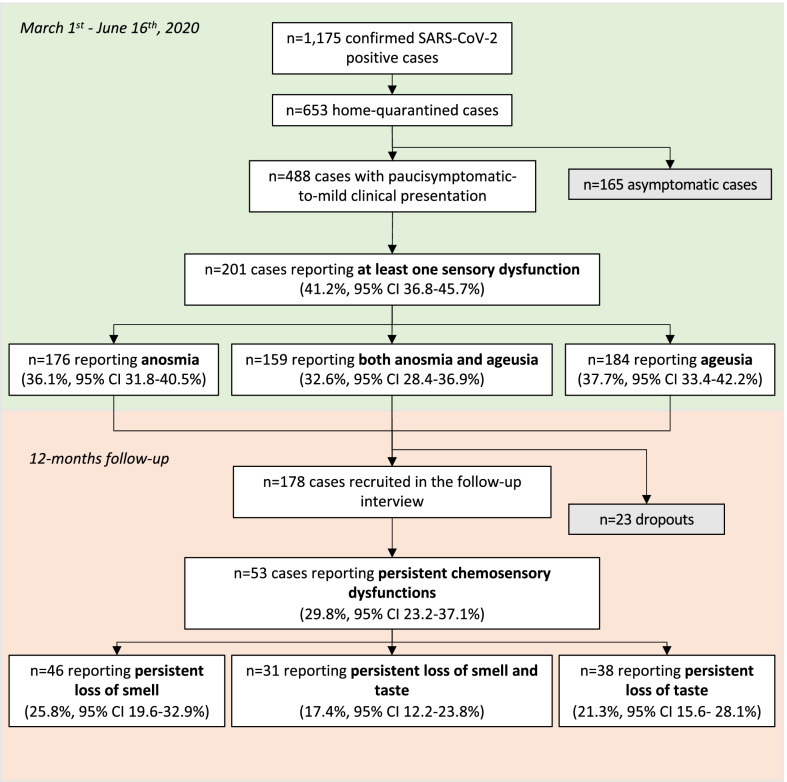
Flowchart of the study of self-reported olfactory and gustatory dysfunctions in COVID-19 cases. District of Foggia (Apulia region, Italy), March 1st, 2020—June 16th, 2021

From March 1st to June 16th, 2020, a total of 1175 COVID-19 confirmed cases were notified in the Foggia district, of which 653 (mean age: 42.9 ± 17.9 years, median age: 45 years, IQR 44–47 years; 53.9% females) were home-quarantined. Clinical presentation of COVID-19 was classified as paucisymptomatic in 55.6% (n = 363) of cases and mild in 19.1% (n = 125) of cases; 165 (25.3%) cases were classified as asymptomatic (Fig. 1, Table 1). The mean estimated incubation period was 9.6 ± 9.8 days (median: 7 days, IQR 3–13.5 days). As of June 16th, 2020, 94.5% (n = 617) of cases were fully recovered with complete viral clearance (mean time to viral clearance: 28 ± 11.9 days; median: 25 days, IQR 18–38 days).

**Table 1:** Demographic and clinical characteristics of home-quarantined COVID-19 cases. District of Foggia (Apulia region, Italy), March 1st—June 16th, 2020

	**Cases (n = 653)**	
**Sex**	n	%
Male	301	46.1
Female	352	53.9
Age (years)		
Mean (SD)	42.9 (17.9)	
Median (IQR)	45 (44–47)	
Age groups		
0–9	28	4.3
10–19	44	6.7
20–29	86	13.2
30–39	103	15.8
40–49	136	20.8
50–59	147	22.5
60–69	73	11.2
70–79	26	3.9
80–89	9	1.4
≥ 90	1	0.2
Comorbidity		
None	536	82.1
At least one comorbidity	117	17.9
Clinical presentation		
Asymptomatic	165	25.3
Paucisymptomatic	363	55.6
Mild	125	19.1

*SD* Standard Deviation, *IQR* Interquartile Ranges

### Baseline prevalence of self-reported olfactory and gustatory dysfunctions

Among the 488 cases with paucisymptomatic-to-mild clinical presentation, 41.2% (n = 201, 95% CI 36.8–45.7%) reported at least one sensory dysfunction. In particular, 36.1% (n = 176, 95% CI 31.8–40.5%) reported anosmia, 37.7% (n = 184, 95% CI 33.4–42.2%) reported ageusia and 32.6% (n = 159, 95% CI 28.4–36.9%) reported both sensory dysfunctions (Fig. 1). Olfactory or gustatory loss was an isolated symptom in 26.8% (n = 131, 95% CI 22.9–30.8%) of cases; namely, only anosmia was reported in 5.5% (n = 27, 95% CI 3.7–7.9%) of cases whereas only ageusia in 3.9% (n = 19, 95% CI 2.4–6.01%) of cases. The co-occurrence of at least one sensory dysfunction and other COVID-19 symptoms was described by 34.8% (n = 70, 95% CI 28.3–41.9%) of cases, with chemosensory dysfunctions preceding the onset of the other COVID symptoms in 10% (n = 7) of cases.

Ageusia, but not anosmia, was significantly more prevalent in females than in males (42% vs. 32.6%, p < 0.05) and related to a slower viral clearance (30.3 ± 13.5 vs. 27.1 ± 11.3 days, p < 0.05; Additional file 1 and Additional file 2). No significant association was observed between self-reported presence of olfactory and gustatory dysfunctions and age, underlying comorbidities, and clinical presentation (Additional file 2).

### Evolution of self-reported olfactory and gustatory dysfunctions

Among the 201 cases that had reported at least one sensory dysfunction, 178 (88.5%) agreed to participate in the follow-up telephone survey (23 [11.4%] dropouts).

After 12 months, 53 cases still reported partial chemosensory dysfunctions (29.8%, 95% CI 23.2–37.1%). Particularly, loss of smell persisted in 25.8% (n = 46, 95% CI 19.6–32.9%) and loss of taste in 21.3% (n = 38, 95% CI 15.6–28.1%) of cases; dysfunctions of both olfactory and gustatory sense were reported in 17.4% (n = 31, 95% CI 12.2–23.8%) of cases (Fig. 1).

Complete resolution of olfactory and gustatory dysfunctions was reported in 70.2% (n = 125, 95% CI 63.5–76.9%) of the respondents in the follow-up interview. The mean duration was 116.6 ± 150.6 days (median 30 days, IQR 13–365 days) for olfactory dysfunction and 104.4 ± 142.3 days (median 30 days, IQR 10–120 days) for gustatory dysfunction. No correlation was observed between the mean duration of chemosensory symptoms and any baseline patients’ demographic and clinical characteristics or the clinical presentation (Table 2).

**Table 2:** Duration (days) of self-reported olfactory and gustatory dysfunctions within 12 months follow-up of COVID-19 cases. District of Foggia (Apulia region, Italy), March 1st, 2020—June 16th, 2021

	Olfactory dysfunction		Gustatory dysfunction	
Sex	Mean time (SD)	p value	Mean time (SD)	p value
Male	100.9 (143.8)	0.1357	102.8 (144.0)	0.4500
Female	126.3 (154.9)		105.5 (141.7)	
Age groups				
≥ 45 years	128.5 (158.1)	0.1361	116.5 (151.7)	0.1289
< 45 years	103.6 (142.5)		92.3 (131.9)	
Comorbidity				
None	117.2 (151.6)	0.4112	103.3 (141.6)	0.4117
At least one comorbidity	110.3 (147.5)		109.8 (148.3)	
Clinical presentation				
Paucisymptomatic	117.8 (151.6)	0.4056	104.3 (142.3)	0.4914
Mild	111.9 (149.6)		104.8 (143.5)	

*SD* Standard Deviation

**Fig. 2:**
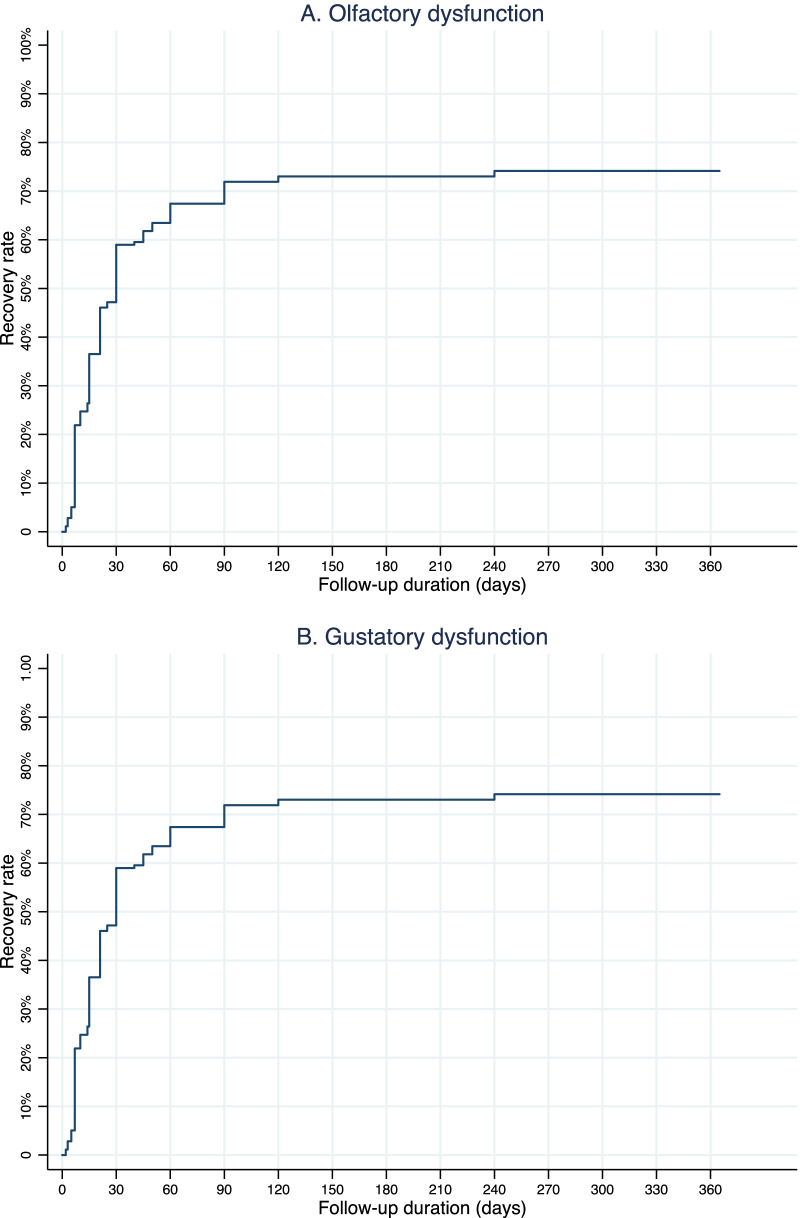
Kaplan–Meier estimates showing the rate of complete recovery of self-reported olfactory **A** and gustatory **B** dysfunctions in COVID-19 cases. District of Foggia (Apulia region, Italy), March 1st, 2020—June 16th, 2021

The rates of full recovery of the sense of smell increased over time, from 59% at 30 days to 67.4% at 60 days and 71.9% at 90 days (Fig. 2a). The rates of full recovery of the sense of taste increased from 61.3% at 30 days to 69.7% at 60 days and 74.7% at 90 days (Fig. 2b). No association was observed between the complete recovery of sensory dysfunctions and any baseline demographic and clinical characteristics or the clinical presentation (Additional file 3).

## Discussion

Olfactory and gustatory dysfunctions are widely reported by COVID-19 patients [4–12] and are emerging as one of the most frequent long-term sequelae of SARS-CoV-2 infection [21–29]. Although chemosensory disorders recover spontaneously in the short-term period in most cases [25, 36, 37], a significant portion of SARS-CoV-2 infected patients continues to suffer from persisting symptoms long after having recovered from the acute illness [30, 38, 39]. The persistence of these symptoms after recovery commonly lasts for 2–3 weeks but may sometimes exceed 6 months. Therefore, the long-term persistence (i.e., six months after clinical onset) of such symptoms deserves to be assessed and investigated. Additionally, considering the large number of people affected by COVID-19 worldwide and the negative impact of anosmia and ageusia on the quality of life [31, 40–43], it is important to quantify the extent and duration of these symptoms to develop effective interventions able to manage the disorders in subjects with SARS-CoV-2 infection.

Our study was conducted on a cohort of confirmed SARS-CoV-2 positive patients with paucisymptomatic or mild disease who did not require hospitalization and were managed by the community healthcare system. At baseline, olfactory or gustatory dysfunctions were present in more than 40% of cases, in agreement with several studies [44–46] and meta-analyses [47, 48]. A higher prevalence of anosmia and dysgeusia symptoms have been reported in studies that included severe cases requiring hospitalization (74.9 and 81.3% of cases, respectively) [12, 49] or healthcare professionals [12].

We performed a prospective evaluation of patients with self-reported gustatory and olfactory dysfunctions. Of the 201 participants included in our study, 178 agreed to participate in the follow-up phase, and only 23 withdrew. Although the majority of the participants (approximately 7 out of 10) in the follow-up reported complete resolution of the olfactory and gustatory disorders after 12 months, a substantial proportion of cases (approximately 3 out of 10) was still complaining of these symptoms. This proportion is remarkable, mainly because our data refer to a population with mild COVID-19. Few other studies comparable to ours in terms of sample size, demographic or clinical characteristics, adherence rates to the follow-up phase, have exceeded a 6-month follow-up and have investigated the recovery of olfactory and gustatory functions beyond this period [21–23, 27, 28]. Of these, only two [22, 27] had a larger sample size at baseline and included a higher number of participants in the follow-up phase.

Most available studies used online questionnaires or telephone interviews for the follow-up phase to collect general and specific clinical information on chemosensory disorders. As opposed to other studies [27, 28], we did not conduct intermediate follow-up surveys. However, we collected data on the persistence and evolution of chemosensory dysfunctions through specific questions (persistence/recovery). These specific questions were administered together with the SNOT-22 test which, in turn, represents a validated subjective method to explore their severity [34, 35].

The rate of persistence of self-reported olfactory and gustatory dysfunctions observed in our study (29.8%) falls within the range reported by available studies (16 to 48%). In particular, Nguyen et al. [23] found that the persistence rate of chemosensory disorders was 24% at 7 months, Nehme et al. [27] reported 16.8% from 7 to 9 months, and Biadsee et al. [21] observed that smell and taste dysfunctions persist respectively in 48% and 38.5% of patients after a mean follow-up of 229 days. Boscolo-Rizzo et al. [22], whose study was similar to ours in design and length of follow-up, estimated that the prevalence of self-reported COVID-19 associated chemosensory dysfunctions was 21.3% at 12 months, roughly in line with our result. Only Renaud et al. [28], who used an objective measurement, described a higher rate of smell recovery (96.1%) at one year.

The reason for the differences between the studies are still unclear and might be of various nature. Primarily, the estimated prevalence may be affected by the methodology used for the data collection. Indeed, it has been proven that the proportion of sensory dysfunctions is affected by the detection instruments, being higher with the use of objective measurements [48, 50]. On the contrary, the assessment of chemosensory dysfunctions based on anamnestic data may fail to detect a considerable proportion of cases [7].

Two very recent case–control studies have evaluated the persistence of smell and taste disorders at least one year after the onset of COVID-19 using psychophysical tests [51, 52]. More specifically, Boscolo-Rizzo et al. estimated that, overall, 58% of cases vs. 18% of controls had an olfactory or gustatory dysfunction, while 33% of cases self-reported a persistently altered sense of smell or taste at the time of the psychophysical evaluation [51]. Vaira et al. found that 26.5% of cases vs. 3.5% of controls had olfactory dysfunction on psychophysical tests, while 25.9% of patients self-reported some form of persistent olfactory loss, suggesting that qualitative and quantitative disturbances of smell may have similar clinical implications on the quality of life [52].

We did not find any baseline demographic or clinical predictive factors for the persistence of symptoms. Interestingly, the occurrence of the syndrome defined “Long COVID” has been reported independently of the severity of the acute phase, being present also in patients with a mild disease [30, 38, 39].

The recovery time is putatively dependent on the underlying pathophysiological mechanisms of sensory dysfunctions. These mechanisms were not investigated in our study but can hopefully be clarified with further purposely designed researches. In our analysis, patients were also asked to estimate the approximate duration of their symptoms. It emerged that a complete recovery of smell and taste tended to occur mostly in the first month after the acute phase of COVID-19. This is in agreement with Nguyen et al. [23], who documented the recovery of the sense of smell and taste mainly during the first 6 weeks after onset using a retrospective questionnaire, and with Nehme et al. [27], who performed an interim interview at 30–45 days after COVID-19 diagnosis.

Olfactory and gustatory dysfunctions are generally considered less disabling and life-threatening than other sensory impairments. Thus, they may not receive adequate medical attention despite their impact on quality of life, especially if prolonged over months. However, olfactory and gustatory deprivations of various cause have been documented as disruptive on daily activities already before the spread of SARS-CoV-2 [31, 40], and their negative impact has been recently confirmed by properly designed studies [41–43]. It is apparent that COVID-19 patients with loss of smell and taste experience a reduced quality of life related to those situations or functions in which the chemical senses play a significant role. Furthermore, they also present with higher levels of psychological distress. In particular, the presence of chemosensory dysfunctions is positively correlated to anxiety and depression, which are attributable to a sense of frustration as the patient often struggles to be fully understood [43].

Our study has some limitations. The self-reported assessment of the severity of symptoms and the retrospective investigation of their persistence within the 12-months follow-up period could be inaccurate due to a recall bias. Moreover, we did not record any possible pharmacological or rehabilitative intervention that could potentially affect the evolution of the olfactory and gustatory dysfunctions during the study period. As previously discussed, another possible limitation lies in the fact that, in our study, chemosensory dysfunctions were not assessed using objective examinations, such as psychophysical and electrophysiological tests, but only on a subjective basis. Therefore, underreporting of chemosensory dysfunctions cannot be excluded. However, in the perspective of a clinical approach taking into account the quality of life, a subjective evaluation may be more significant than an objective assessment of the disorders. Moreover, due to the absence of a control group, our findings could have been biased by the underlying prevalence of chemosensory dysfunctions in the general population [53, 54]. Also the inclusion of only patients with paucisymptomatic-to-mild COVID-19 may have influenced both the prevalence of sensory disorders and the characteristics or the timing of their evolution. Finally, the possibility of spontaneous recoveries of a post-viral olfactory loss even beyond one year from the onset cannot be excluded [55].

## Conclusions

Olfactory and gustatory dysfunctions persist in a substantial portion of patients with previous paucisymptomatic-to-mild clinical COVID-19 up to 12 months after disease onset. As SARS-CoV-2 infection may be associated with long-term smell and/or taste loss that may significantly impact the quality of life, there is a need for a better understanding of the extent and duration of these symptoms. In addition, a better understanding of the physiological mechanisms driving the recovery is required to facilitate the development of effective preventive and therapeutic strategies.

## Supplementary Information

**Additional file 1:** Univariate analysis of variables associated with self-reported chemosensory dysfunctions in COVID-19 cases. District of Foggia (Apulia region, Italy), March 1st - June 16th, 2020.

**Additional file 2:** Multivariate analysis of variables associated with self-reported chemosensory dysfunctions in COVID-19 cases. District of Foggia (Apulia region, Italy), March 1st - June 16th, 2020.

**Additional file 3:** Multivariate analysis of variables associated with complete recovery of self-reported olfactory and gustatory dysfunctions in COVID-19 cases. District of Foggia (Apulia region, Italy), March 1st, 2020–June 16th, 2021.

## Data Availability

The authors declare that the data supporting the findings of this study are available within the article and its Additional file 1, Additional file 2, and Additional file 3.
