# Supplementary material for: Self-reported olfactory and gustatory dysfunctions in COVID-19 patients: a 1-year follow-up study in Foggia district, Italy
Source: BMC Infect Dis. 2022 Jan 22;22:77. doi: 10.1186/s12879-022-07052-8 (PMC8783175; doi:10.1186/s12879-022-07052-8)
Supplement: Supplementary file 1 — Additional file 1: Univariate analysis of variables associated with self-reported chemosensory dysfunctions in COVID-19 cases. District of Foggia (Apulia region, Italy), March 1st - June 16th, 2020. [file 12879_2022_7052_MOESM1_ESM.pdf]

Additional file 1. Univariate analysis of variables associated with self-reported chemosensory dysfunctions in COVID-19 cases. District of Foggia (Apulia region, Italy), March 1st - June 16th, 2020

	Olfactory dysfunction				Gustatory dysfunction				At least one sensory dysfunction			
	Yes	No	OR [95% CI]	p value	Yes	No	OR [95% CI]	p value	Yes	No	OR [95% CI]	p value
**Sex**												
Male, n (%)	71 (31.7)	153 (68.3)	0.70 [0.47-1.03]	0.0641	73 (32.6)	151 (67.4)	0.66 [0.45-0.98]	0.0317	84 (37.5)	140 (62.5)	0.75 [0.51-1.10]	0.1273
Female, n (%)	105 (39.8)	159 (60.2)			111 (42.0)	153 (58.0)			117 (44.3)	147 (55.7)		
**Age (years)**												
Mean (SD)	43.5 (14.7)	42.1 (18.8)	-	0.1967	43.5 (14.6)	42.1 (18.9	-	0.1955	43.4 (14.7)	42.0 (19.1)	-	0.1904
Median (IQR)	45 (32-53)	44 (28-56)	-	-	45 (32-55)	44 (28-56)	-	-	45 (32-55)	44 (28-56)	-	-
**Age groups**												
≥45 years, n (%)	90 (37.3)	151 (62.7)	1.11 [0.75-1.64]	0.5612	94 (39.0)	147 (61.0)	1.11 [0.76-1.63]	0.5586	102 (42.3)	139 (57.7)	1.09 [0.75-1.59]	0.6148
<45 years, n (%)	86 (34.8)	161 (65.2)			90 (36.4)	157 (63.6)			99 (40.1)	148 (59.9)		
**Comorbidity**												
None, n (%)	148 (37.1)	251 (62.9)	0.78 [0.46-1.30]	0.3171	153 (38.4)	246 (61.6)	0.86 [0.51-1.42]	0.5362	170 (42.6)	229 (57.4)	0.72 [0.43-1.19]	0.1778
At least one comorbidity, n (%)	28 (31.5)	61 (68.5)			31 (34.8)	58 (65.2)			31 (34.8)	58 (65.2)		
**Clinical presentation**												
Paucisymptomatic, n (%)	126 (34.7)	237 (65.3)	1.25 [0.80-1.94]	0.2882	128 (35.3)	235 (64.7)	1.49 [0.96-2.29]	0.0577	141 (38.8)	222 (61.2)	1.43 [0.94-2.23]	0.0728
Mild, n (%)	50 (40.0)	75 (60.0)			56 (44.8)	69 (55.2)			60 (48.0)	65 (52.0)		
**Incubation period (days)**												
Mean (SD)	8.9 (8.0)	9.4 (10.3)	-	0.3169	8.8 (7.7)	9.5 (10.5)	-	0.2395	8.7 (7.7)	9.7 (10.7)	-	0.1492
**Time to viral clearance (days)**												
Mean (SD)	29.1 (12.7)	28.5 (12.1)	-	0.3090	30.3 (13.5)	27.7 (11.3)	-	0.0117	29.7 (13.4)	28 (11.5)	-	0.0690

OR: Odds Ratio; CI: Confidence Interval; SD: Standard Deviation; IQR: Interquartile Ranges
