# Supplementary material for: Self-reported olfactory and gustatory dysfunctions in COVID-19 patients: a 1-year follow-up study in Foggia district, Italy
Source: BMC Infect Dis. 2022 Jan 22;22:77. doi: 10.1186/s12879-022-07052-8 (PMC8783175; doi:10.1186/s12879-022-07052-8)
Supplement: Supplementary file 2 — Additional file 2: Multivariate analysis of variables associated with self-reported chemosensory dysfunctions in COVID-19 cases. District of Foggia (Apulia region, Italy), March 1st - June 16th, 2020. [file 12879_2022_7052_MOESM2_ESM.pdf]

Additional file 2. Multivariate analysis of variables associated with self-reported chemosensory dysfunctions in COVID-19 cases. District of Foggia (Apulia region, Italy), March 1st - June 16th, 2020

	Olfactory dysfunction		Gustatory dysfunction		At least one sensory dysfunction	
	**OR [95% CI]**	**p value**	**OR (95% CI)**	**p value**	**OR (95% CI)**	**p value**
**Sex**<br>Male vs. female	0.69<br>[0.48-1.01]	0.057	0.65<br>[0.45-0.95]	0.027	0.74<br>[0.51-1.06]	0.106
**Age groups**<br>≥45 years vs. <45 years	1.16<br>[0.79-1.71]	0.448	1.12<br>[0.76-1.64]	0.563	1.14<br>[0.78-1.67]	0.477
**Comorbidity**<br>None vs. at least one comorbidity	0.70<br>[0.42-1.17]	0.178	0.77<br>[0.46-1.27]	0.304	0.64<br>[0.39-1.06]	0.084
**Clinical presentation**<br>Paucisymptomatic vs. mild	1.27<br>[0.84-1.95]	0.256	1.52<br>[0.99-2.31]	0.050	1.49<br>[0.98-2.27]	0.057

OR: Odds Ratio; CI: Confidence Interval
