# Supplementary material for: Self-reported olfactory and gustatory dysfunctions in COVID-19 patients: a 1-year follow-up study in Foggia district, Italy
Source: BMC Infect Dis. 2022 Jan 22;22:77. doi: 10.1186/s12879-022-07052-8 (PMC8783175; doi:10.1186/s12879-022-07052-8)
Supplement: Supplementary file 3 — Additional file 3: Multivariate analysis of variables associated with complete recovery of self-reported olfactory and gustatory dysfunctions in COVID-19 cases. District of Foggia (Apulia region, Italy), March 1st, 2020–June 16th, 2021. [file 12879_2022_7052_MOESM3_ESM.pdf]

Additional file 3. Multivariate analysis of variables associated with complete recovery of self-reported olfactory and gustatory dysfunctions in COVID-19 cases. District of Foggia (Apulia region, Italy), March 1st, 2020 - June 16th, 2021

	Olfactory dysfunction				Gustatory dysfunction				At least one sensory dysfunction			
**Sex**	**Yes**	**No**	**OR [95% CI]**	**p value**	**Yes**	**No**	**OR [95% CI]**	**p value**	**Yes**	**No**	**OR [95% CI]**	**p value**
Male, n (%)	16 (34.8)	56 (42.4)	1.32	0.433	16 (42.1)	56 (40.0)	0.87	0.723	14 (45.2)	58 (39.4)	1.41	0.316
Female, n (%)	30 (65.2)	76 (57.6)	[0.65-2.68]		22 (57.9)	84 (60.0)	[0.42-1.83]		17 (54.8)	89 (60.5)	[0.71-2.79]	
**Age groups**												
≥45 years, n (%)	27 (58.7)	62 (47.0)	0.60	0.157	23 (60.5)	66 (47.1)	0.57	0.151	18 (58.1)	71 (48.3)	0.54	0.080
<45 years, n (%)	19 (41.3)	70 (53.0)	[0.29-1.21]		15 (39.5)	74 (52.9)	[0.27-1.22]		13 (41.9)	76 (51.7)	[0.28-1.07]	
**Comorbidity**												
None, n (%)	39 (84.8)	110 (83.3)	1.29	0.595	31 (81.6)	118 (84.3)	0.98	0.970	26 (83.9)	123 (83.7)	1.11	0.816
At least one comorbidity, n (%)	7 (15.2)	22 (16.7)	[0.49-3.41]		7 (18.4)	22 (15.7)	[0.37-2.62]		5 (16.1)	24 (16.3)	[0.45-2.74]	
**Clinical presentation**												
Paucisymptomatic	33 (71.7)	93 (70.5)	1.06	0.870	27 (71.1)	99 (70.7)	1.09	0.827	22 (80.0)	104 (70.7)	1.09	0.811
Mild	13 (28.3)	39 (29.5)	[0.49-2.28]		11 (28.9)	41 (29.3)	[0.49-2.45]		9 (20.0)	43 (29.3)	[0.52-2.27]	

OR: Odds Ratio; CI: Confidence Interval
